# Menstrual Psychosis with Premenstrual Onset: A case presentation

**DOI:** 10.1192/j.eurpsy.2024.1544

**Published:** 2024-08-27

**Authors:** S. Turkan, E. Kara, R. S. İlhan, S. Yalcın Sahiner, M. C. Saka

**Affiliations:** ^1^Psychiatry; ^2^Child and Adolescent Psychiatry, Ankara University, Ankara, Türkiye

## Abstract

**Introduction:**

Menstrual psychosis has an acute onset and is characterised by confusion, stupor and mutism, delusions, hallucinations, or a manic syndrome lasting for a brief duration, with full recovery. These symptoms maintain periodicity in rhythm with the menstrual cycle. The symptoms may appear in the premenstrual phase or may begin with the onset of menstrual flow (catamenial psychoses). Usually, menstrual psychosis has a polymorphism of both psychotic and affective symptoms (Brockington I. Menstrual psychosis. *World Psychiatry.* 2005;4(1):9-17.). In this article we present a case of menstrual psychosis with premenstrual onset.

**Objectives:**

A 26 years old, nulliparous single female with one mentally ill relative on her mother’s side (her grandmother) presented with an episodic illness characterized by mood swings, irrelevant speech, irritability, suspiciousness and thought disorder related to her menstrual cycles. She had earlier suffered prolonged attacks of mania, developed a recurrent episodic illness which returned every month for five years. Her menses began at 15. She presented with the history of a few episodes of manic illness starting five days before and ending suddenly with the onset of the menses.

**Methods:**

On mental status evaluation during the index episode, the patient was agitated, had labile affect, grandiose and referential delusions and erotomania. A detailed physical examination, routine biochemistry, and gonadal hormonal assay were unremarkable.

**Results:**

She was started on olanzapine 10 mg/day, lithium 1200 mg/day and low-dose clonazepam. Although the severity of the psychotic and affective symptoms gradually reduced during the future menstrual cycles, they did not completely resolve.

**Conclusions:**

The pathophysiology of menstrual psychosis is not exactly understood, but it has been postulated that fluctuation of the sex hormones occurring during the menstrual cycle is responsible. Previous studies have reported the association of psychosis with estrogen withdrawal (Mahé V, Dumaine A. Oestrogen withdrawal associated psychoses. *Acta Psychiatr Scand.* 2001;104(5):323-331.). Treatment strategies for menstrual psychosis include the use of oral contraceptive pills for the regulation of hormones during the menstrual cycle, in our case patient did not want to use oral contraceptive pills.

**Disclosure of Interest:**

None Declared

